# Summary of the ISEV workshop on extracellular vesicles as disease biomarkers, held in Birmingham, UK, during December 2017

**DOI:** 10.1080/20013078.2018.1473707

**Published:** 2018-05-17

**Authors:** Aled Clayton, Dominik Buschmann, J. Brian Byrd, David R. F. Carter, Lesley Cheng, Carolyn Compton, George Daaboul, Andrew Devitt, Juan Manuel Falcon-Perez, Chris Gardiner, Dakota Gustafson, Paul Harrison, Clemens Helmbrecht, An Hendrix, Andrew Hill, Andrew Hoffman, Jennifer C. Jones, Raghu Kalluri, Ji Yoon Kang, Benedikt Kirchner, Cecilia Lässer, Charlotte Lawson, Metka Lenassi, Carina Levin, Alicia Llorente, Elena S. Martens-Uzunova, Andreas Möller, Luca Musante, Takahiro Ochiya, Ryan C Pink, Hidetoshi Tahara, Marca H. M. Wauben, Jason P. Webber, Joshua A. Welsh, Kenneth W. Witwer, Hang Yin, Rienk Nieuwland

**Affiliations:** a Tissue Microenvironment Group, School of Medicine, Cardiff University, Cardiff, UK; b Division of Animal Physiology and Immunology, Technical University of Munich and Institute of Human Genetics, University Hospital, LMU Munich, Munich, Germany; c Department of Internal Medicine, University of Michigan, 5570C MSRB II, Ann Arbor, USA; d Department of Biological and Medical Sciences, Oxford Brookes University, Oxford, UK; e Department of Biochemistry and Genetics, La Trobe Institute for Molecular Science, La Trobe University, Melbourne, Victoria, Australia; f SkySong Center for Innovation, Arizona State University, Scottsdale, AZ, USA; g nanoView Diagnostics, Boston, MA, USA; h School of Life & Health Sciences, Aston University, Birmingham, UK; i Exosomes Laboratory & Metabolomics Platform, CIC bioGUNE, CIBEREHD, IKERBASQUE Research Foundation, Derio, Spain; j Research Department of Haematology, Haemostasis Research, University College London, London, UK; k Department of Laboratory Medicine and Pathobiology, University of Toronto, Toronto, Canada; l Institute of Inflammation and Ageing, College of Medical and Dental Sciences, University of Birmingham, Birmingham, UK; m Particle Metrix GmbH, Meerbusch, Germany; n Laboratory of Experimental Cancer Research, Department of Radiation Oncology and experimental Cancer Research, Ghent University, Ghent, Belgium; and Cancer Research Institute Ghent, Ghent, Belgium; o Regenerative Medicine Laboratory, Cummings School of Veterinary Medicine, Tufts University, North Grafton, MA, USA; p Department of Vaccine Branch, National Cancer Institute, Bethesda, MD, USA; q Department of Cancer Biology, Metastasis Research Center, UT MD Anderson Cancer Center, Houston, TX, USA; r Korea Institute of Science and Technology, Center for Bio-microsystems, Seoul, S. Korea; s Division of Animal Physiology and Immunology, TUM School of Life Sciences Weihenstephan, Technical University of Munich, Munich, Germany; t Institute of Medicine at Sahlgrenska Academy Krefting Research Centre University of Gothenburg, Gothenburg, Sweden; u Department of Comparative Biomedical Sciences, Royal Veterinary College Royal College Street, London, UK; v Institute of Biochemistry, University of Ljubljana, Ljubljana, Slovenia; w Afula and The Bruce Rappaport Faculty of Medicine, Emek Medical Center, Technion, Haifa, Israel; x Department of Molecular Cell Biology, Oslo University Hospital, Oslo, Norway; y Department of Urology, Erasmus MC, Rotterdam, CA, The Netherlands; z Tumour Microenvironment Laboratory, Tumour Microenvironment Laboratory, QIMR Berghofer Medical Research Institute, Herston, Australia; aa Department of Medicine, Division of Nephrology, University of Virginia, Charlottesville, VA, USA; ab Division of Molecular and Cellular Medicine, National Cancer Center Research Institute, Tokyo, Japan; ac Department of Cellular and Molecular Biology, Institute and Graduate School of Biomedical & Health Sciences, Hiroshima University, Hiroshima City, Japan; ad Faculty of Veterinary Medicine, Dept. Biochemistry & Cell Biology, Utrecht University, Utrecht, The Netherlands; ae Department of Molecular and Comparative Pathobiology, Johns Hopkins Institute for NanoBio Technology, Johns Hopkins University, Baltimore, USA; af School of Pharmaceutical Sciences, Tsinghua University, Beijing, China; ag Department Laboratory Experimental Clinical Chemistry, Academic Medical Center, University of Amsterdam, DE, Amsterdam, The Netherlands

**Keywords:** Biomarkers, extracellular vesicles, exosomes, cancer, serum, plasma, urine

## Abstract

This report summarises the presentations and activities of the ISEV Workshop on extracellular vesicle biomarkers held in Birmingham, UK during December 2017. Among the key messages was broad agreement about the importance of biospecimen science. Much greater attention needs to be paid towards the provenance of collected samples. The workshop also highlighted clear gaps in our knowledge about pre-analytical factors that alter extracellular vesicles (EVs). The future utility of certified standards for credentialing of instruments and software, to analyse EV and for tracking the influence of isolation steps on the structure and content of EVs were also discussed. Several example studies were presented, demonstrating the potential utility for EVs in disease diagnosis, prognosis, longitudinal serial testing and stratification of patients. The conclusion of the workshop was that more effort focused on pre-analytical issues and benchmarking of isolation methods is needed to strengthen collaborations and advance more effective biomarkers.

## Introduction

The potential utility for extracellular vesicles (EVs) as disease biomarkers has attracted unprecedented commercial and academic interests over the last decade. The ability to identify disease-related EVs within body fluids is compelling for cancer, metabolic and cardiovascular disease, neurodegenerative conditions and other disease syndromes. However, the complexity of biofluids, competing technologies and the nano-scale nature of EVs present challenges that we need to face before EV biomarkers can be routinely applied. We therefore held an ISEV workshop during 13–14 December 2017, at the Medical School, University of Birmingham, UK, to discuss critical issues, and to identify potential approaches to enable better collaboration and greater success in advancing effective biomarkers.

**Figure 1.**
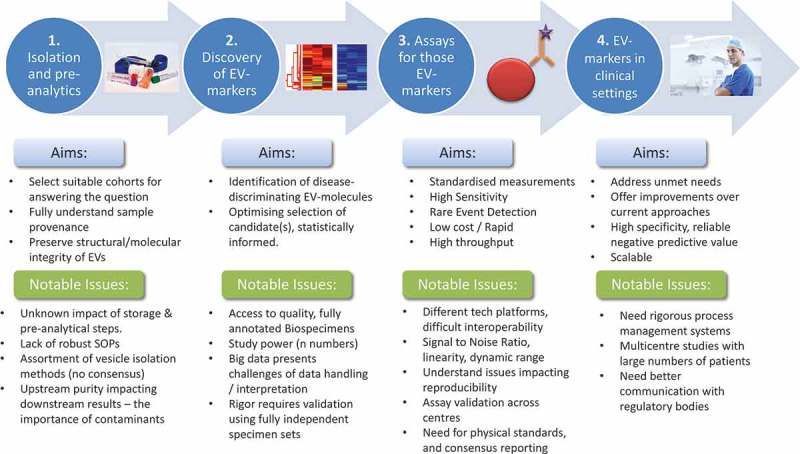
Workshop topic areas and some highlighted problems.

ISEV members submitted 73 abstracts, and 60 researchers were invited to contribute as presenters and/or in round table discussions. Talks included plenary presentations from recognised EV research leaders and invited perspectives from outside the EV realm. Selections took account of gender, career stage balance and representation from all geographical chapters, and the workshop was structured from bench to bedside [1]: Pre-analytical variables, biobanking and vesicle isolation [2], discovery of EV biomarkers [3], systems/assays to detect EV-markers (rare event analyses) and [4] taking EV assays to the clinic. We summarise presented viewpoints, achievements and highlighted problems we face for developing EV-based biomarkers (Figure 1).

## General perspectives on biomarkers

Carolyn Compton from the (U.S.) National Biomarker Development Alliance (NBDA) (Arizona State University, USA) presented an excellent overview of the biomarker field from outside the EV realm. For 21st century medicine, there is the expectation that the molecular profiles of biospecimens will facilitate early disease detection, diagnosis and personalised medicine.

Enabling technologies in the life sciences have dramatically increased assay sensitivity and evolved exponentially, resulting in a “Tsunami of new data”, that allow previously unimaginable deep-characterisation of biospecimens, such as next-generation sequencing. As the amount of data escalate, so do the challenges in handling, analysing and understanding the arising datasets. We are currently unclear about how we can fully capture this information to aid the identification of biomarkers and maximise their utility in clinical settings.

In marked contrast to the explosion of data generating tools, there remains a massive attrition in terms of developing new therapeutics with on average 1:10,000 chance of success, which continues to be slow, typically taking over 12 years, and costly, 2–5 billion USD, before a final and approved marketable product is achieved. A major reason for this slow and costly process is the lack of validated biomarkers, highlighting the urgent need for new technologies to address this need. Although around 150,000 biomarkers have been claimed in the scientific literature, the actual number of biomarkers that are routinely used in clinical settings is only around 100 [1], although this figure may have changed since 2011. The NBDA has examined the principal deficiencies that have led to a general failure in biomarker discovery and application, detailed in Table 1.

**Table 1.** NBDA top ten reasons for the failure in developing effective biomarkers [2].

1	Poor access to rigorously annotated, fit-for-purpose biospecimens from stringently phenotyped sources
2	Insufficient control of pre-analytical parameters
3	Low reproducibility of academic publications
4	Incomplete understanding of physiology
5	Variable analytical standards
6	Idiosyncratic laboratory-specific analytical methods
7	Small studies lacking statistical power
8	Chaotic data reporting formats and poor database interoperability
9	Poor compliance on reporting standards by scientific journals
10	Poor to non-existent quality management systems

Medical sciences grossly suffer from a lack of scientific reproducibility. For example, a recent study highlighted that of 53 studies published in high-impact journals in oncology and haematology, only 11% could be reproduced [3]. The actual extent of the irreproducibility problem in biomedical research, in general, and particularly in biomarker research is currently unknown and is under investigation. It is expected to be significant because independent validation of research findings prior to publication is extremely rare. Few biomedical researchers routinely validate their findings in their own laboratories, and even fewer seek external validation of their results in independent laboratories. Importantly, from a biomarker point of view, few studies attend to the quality and consistency of human biospecimens that are the sources of the analytes they are measuring and the true foundation of all biomarker research efforts. The implication being that the existing scientific literature based on human biospecimen analysis is, at best, highly problematic and likely reporting unreliable results. Consequently, with such paucity of rigour in terms of biospecimens collection and handling, it is no surprise there is a general failure in biomarker discovery.

The modality of specimen collection has the capacity to artificially alter the molecular integrity and/or the composition of biospecimens, thereby changing the analysis data. Thus, pre-analytical variables have the capacity to exert a major impact on all subsequent information being gathered from the biospecimen and artefactually alter multiple types of analysis data. For example, there are numerous reports of collection, processing and handling variables that affect the composition of blood and serum. Many of these have been demonstrated to have a significant impact on EV analysis. Unfortunately, the list of pre-analytical variables is long, and includes at least 17 process-related factors that may affect analysis data (Table 2). In addition, there are numerous donor-related variables including age, body mass systemic disorders, etc. about which very little is currently known.

**Table 2.** Factors relevant for biospecimen variability during blood collection/handling/storage.

1. Tourniquet vs. none	2. Tourniquet time
3. Central line or artery vs. peripheral vein	4. Draw order
5. Temperature and duration of storage	6. Tube type
7. Tube volume^a^	8. Tube inversions
9. Vacuum tube, butterfly vs. syringe^a^	10. Type of anticoagulant
11. Type of port (if used for access)	12. Total time of draw
13. Number of centrifugations^a^	14. Needle bore
15. Time to centrifugation^a^	16. Centrifuge speed^a^
17. Tube agitation during transport^a^	

^a^ Indicates where data exist for impact on EVs [4–10].

In many studies, such specific details are not considered, controlled or reported, and hence it is impossible to accrue sufficient evidence to develop optimised standard operating procedures (SOPs) when both analytes and analysis are variable. The study results take centre stage, and pre-analytical details, compliance with protocols and the management of adherence to these are rarely dealt with in a suitably rigorous fashion. In 2013, ISEV published a position paper to create awareness on the impact of pre-analytical variables on EV analysis in body fluids [10]. Recently, the EV-TRACK consortium created a platform (www.evtrack.org) to improve the transparency and compliance in reporting such parameters with the aim to increase reproducibility of EV research, a prerequisite to initiate EV biomarker [11].

Taken together, rigorous biospecimen research is urgently needed to boost the quality of medical sciences in general, and a prerequisite to implement EV-based biomarkers into the clinics. To achieve this goal, SOPs regarding biospecimen collection and handling must be developed as a sound basis for future EV research.

## General perspectives on EV biomarkers

Raghu Kalluri (UT MD Anderson Cancer Center, Houston, USA) showed the potential value of EV-associated DNA. EVs from several human and murine cancer lines contain fragments of genomic DNA, up to 19 kb long and DNase resistant, suggesting an intraluminal encapsulation of the DNA. It clearly established now that the DNA is double stranded and can also be in the single-stranded form. In plasma, most genomic DNA was associated to EVs, and genetic profiling (of EVs) identified tumour-related mutations such as KRAS in pancreatic cancer patients [12]. Importantly, regarding tumour heterogeneity the mutational profile of biopsies from distinct sites within a patient can differ. Sampling circulating EVs, however, potentially captures all such mutations from a single biospecimen, providing a more complete picture of genomic aberrations. Because healthy individuals also exhibited such mutations in their serum EV-associated DNA, this approach may also predict potential propensity towards developing cancer but with cautionary note that mutation detection does not guarantee development of clinical disease of cancer.

EVs with glypican-1 on their surface were previously identified as a biomarker for pancreatic cancer [13]. Since this publication, several different laboratories have validated the concept that glypican-1 is enriched on the EVs derived from cancer cells [14]. Additionally, multiple antibodies have been identified that can recognise glypican-1 on the surface of cancer exosomes [15], and validation studies are currently underway.

These example studies identify broad perspectives for EV utility in cancer as a multiplex set of molecules that inform us about disease status and likely hold predictive value. They also reiterate the importance of analytical methods, and the details therein and an important role for inter-lab validation of such discoveries prior to future evolution towards clinical application.

### Workshop topic 1. EV Isolation and pre-analytical considerations

Rienk Nieuwland (University of Amsterdam, Netherlands) showed that blood contains all blood clotting components apart from one essential component. This is a transmembrane protein, tissue factor (TF), which activates coagulation factor VII to factor VIIa, and thereby triggers the clotting of blood. In physiological conditions, TF is abundantly present on EVs in normal human saliva (and other body fluids such as urine and lacrima), and the ability of EVs from saliva to trigger blood clotting is comparable to snake venom [16].

However, TF-EVs can also be present within peripheral blood, and in various types of cancer, TF-EVs released from the tumour are thought to increase the risk of developing venous thromboembolism (VTE). Recently, a prospective multicentre trial of almost 900 cancer patients was completed to predict VTE using four predication scores [17]. The main conclusion was that none of these scores can be used to identify patients at risk of VTE. But in a subgroup of 650 cancer patients the ability of an EV-based clotting test, the fibrin generation test (FTG), to identify cancer patients at risk of developing VTE was examined. This assay outperformed current predictive models pancreatic cancer patients (*n* = 100). During the study, however, knowledge of blood handling/collection has evolved and the FGT test is amenable to great improvements [18]. Taken together, given the fact that each year about 10,000,000 new cases of VTE are described, of which 20% are associated with cancer, further exploring the coagulant properties of EVs to predict VTE in certain types of cancer, is promising.

Cecilia Lässer (University of Gothenburg, Sweden) addressed the problem of isolating EVs from 0.5–1 mL of plasma/serum, and to separate EVs from lipoproteins and chylomicrons. Lipoprotein particles such as LDL, HDL and chylomicrons resemble EVs when it comes to size and/or density and it can therefore be hard to isolate pure EVs from blood. By combining separation based on density, using an iodixanol cushion, followed by separation based on size, by size exclusion chromatography (SEC), effective isolation of EVs well separated from lipoprotein particles was achieved. This approach also revealed that the majority of events observed in plasma, by techniques such as NTA is most likely lipoprotein particles and not EVs, highlighting the hurdles working with complex clinical samples [19]. A similar approach was described by An Hendrix (Ghent University, Belgium), except reversing the order with SEC followed by density gradient centrifugation. Processing plasma/serum in this fashion is effective but laborious, however, and a more tractable approach would be needed to handle large sample numbers. As patient specimens are precious and often of limited volumes, adapting isolation and/or analysis techniques for small-volume biofluid samples will likely aid in moving EV biomarker candidates into the clinics.

Lesley Cheng (La Trobe University, Australia) emphasised the relevance of pre-analytical standardisation of blood and urine sample collection and handling on EVs, in particular for isolation of EV-associated micro (mi)RNA [20]. Importantly, even when the effect of pre-analytical variables on the sample are unknown, consistency and compliance with downstream SOPs is extremely important to enable the comparison of results between collected samples. For blood collection, the effects of pre-analytical variables and cofounders were discussed, such as the effect of haemolysis, and the preferential use of fasting blood samples [21]. For collection of urine samples, the value of adding protease inhibitors and reducing agents were discussed. For downstream RNA-extraction, a variety of commercial kits was compared, and despite the limitations of such kits the obtained results may be more consistent than traditional ultracentrifugation-based isolation approaches.

Luca Musante (University of Virginia, USA) also presented data on urine collection, preferring first morning urine for collection, and demonstrating that a citrate-based buffer is beneficial in controlling pH and reducing precipitates after thawing, although this may affect size and composition of EVs. Improved control of inter-day variation with regards to EV recovery, was achieved in the presence of protease inhibitors, but the optimal inhibitor cocktail remains unresolved. It was shown that two different protease inhibitor cocktails had different impacts on the ratio of Tamm-Horsfall protein (THP) polymerisation and yield of both THP and urinary EVs in the centrifugation pellets. Reducing agents to breakdown THP polymers and protein aggregates was viewed as advantageous for EV recovery but still minimal variation of the experimental condition such as pH, presence or absence of protease inhibitors can dramatically influence the repartition of EVs and THP in the pellet and supernatant, respectively. However, the physical associations between EVs and THP remains ill defined, and perhaps is an aspect deserving of greater attention.

There is a need of experimental standards to monitor the efficacy of EV-protecting steps and isolation procedures. Being able to spike-in reference EVs, EV-like particles or an artificially synthesised EV mimic was thought to be a valuable approach to identify critical variables in collection, storage and handling of EV-containing samples. An Hendrix described ongoing studies to manufacture an EV-like standard, which is endogenously fluorescent, thus allowing analysis by multiple platforms. Likely, the field may require an assortment of standards, depending on the measurement-platform. The use of artificial EV standards is likely to facilitate the development of evidence-based SOPs for collection, storage and handling of EVs.

Unfortunately, many researchers need to rely on biospecimens from established bio-banking organisations, in which the methods for collection, handling and storage were not intended for EV research. Ryan Pink (Oxford Brookes, UK) highlighted a potential opportunity, involving a UK national initiative. The UK Biobank Consortium, which has been collecting specimens from 500,000 healthy individuals over the last 11 years. The specimens are well annotated, with information on lifestyle, physiology, blood biochemistry and genomics, and about 200,000 individual whole-body MRI scans. Critically, this data is linked to full health records within the UK’s National Health Service, thus offering a comprehensive set of materials and metadata currently untapped for EV studies. However, the release of specimens will be limited in terms of quantity, that is no more than 0.5 mL per individual, and hence our methods for isolation and profiling of EVs need to be compatible with this level of input material. Ryan presented work and discussion on extracting EVs from 50 µl of plasma, the effect of freezing and collecting functional RNA and protein, making small biobank sample use more realistic. Nevertheless, there are opportunities for EV researchers to exploit biobanks, and to integrate EV-profiling information with other available information.

### Workshop topic 2. Profiling EVs from biofluids

Takahiro Ochiya (National Cancer Center Research Institute, Tokyo, Japan) presented a plenary overview of the value of EV profiling in disease, concentrating on an $80 million study to develop miRNA-based technologies for disease diagnoses.

On average, 37% of blood-borne miRNA is present within EVs, and three miRNAs were identified, miR-149-3p, 2861 and 4463, that are consistently present in all serum samples, and which potentially provide an internal normalisation control. The team profiled vesicular miRNA across a spectrum of cancer types, dementia and healthy individuals, accumulating data on over 42,000 individuals. The data were processed by Fishers linear discriminant analysis, in an iterative fashion to identify combinations of candidate miRNAs capable to discriminate health from disease. Many successes of this approach were shown, revealing the extraordinary capacity to diagnose colon cancers using a set of 5 miRNAs with >95% sensitivity and specificity, and similar findings were shown for common cancers such as lung, brain and pancreas cancer.

Results using the ExoScreen assay from the same group [22] showed that detection of CD145/CD9 dual positive cancer EVs is possible using 5 µL serum of colorectal cancer patients, and that EVs positive for GPRC5C/CD63 can discriminate pancreatitis patients from stage II pancreatic cancer. The assay is flexible for other cancer types also and examples of other cancer site-specific bead pairs were presented. There is therefore proven value in examining vesicles in terms of profiling their microRNA, and computational tools will allow optimised biomarker sets to be revealed, and subsequently tested for their diagnostic power.

Juan Manuel Falcon-Perez Perez (CIC bioGUNE, Derio, Spain) highlighted the ongoing revolution in EV research and application. The utility of circulating EVs to assess liver injury was illustrated, showing that alterations in the hepatocyte proteome are partly reflected by the proteome of EVs, thus offering an alternative for liver biopsy. Interestingly, liver injury often leads to changes in liver-specific enzymes, and these remain catalytically active when released within EVs. Addition of hepatocyte EVs to serum results in a host of new metabolites, such as ornithine, due to the catalytic activities of EV-associated arginase [23]. Hence, the assessment of liver injury can be inferred from profiling EVs and the repertoire of metabolites within biofluids [24].

There were several presentations from Juan Manuel Falcon-Perez, Alicia Llorente (Oslo University Hospital, Norway), and Elena Martens-Uzunova (Erasmus MC, Rotterdam, Netherlands) about prostate cancer, using urine as a source of EVs. They showed promising data where EV proteins such as CD10 and Flt1 could discriminate enlarged yet benign prostatic hyperplasia from genuine prostate cancer. Also, there were examples of EV-associated messenger RNA and microRNA, e.g. CDH3, or miR-196a-5p, being consistently downregulated in prostate cancer, and examples of other types of small noncoding RNA such as snoRNA and tRNA detectable in EVs. However, the detection of some of these markers critically depends on the sample processing methodology in terms of both EV capture and RNA extraction, where different commercial kits show enormous differences in RNA yield and quality suitable for downstream applications.

In quite a different disease setting, Metka Lenassi (University of Ljubljana, Slovenia) showed that HIV patients who are apparently virus free, may encounter non-AIDS-related disorders, such as inflammatory-related complications [25]. The utility of EVs was explored to identify potential active HIV reservoirs in aviremic individuals. EVs were isolated from plasma by sucrose cushion/centrifugation that efficiently removes LDL and chylomicrons. With these specimens, the HIV-encoded protein Nef was detected in half of aviremic patients by ELISA. Nef levels associated with the antiretroviral therapy (ART) regimen, but did not correlate with clinical characteristics [26]. Additionally, the profiling of vesicular miRNAs discovered miR-20a and miR-223, showing greater potential, and which correlated with CD4/CD8 T cell ratios. Thus, there is utility in circulating EVs to determine the presence of active HIV reservoirs that may be drivers of co-morbidities even in the absence of infectious HIV virus.

In summary, profiling the complex components of EVs, or EV-induced metabolites hold direct promise as disease indicators across a variety of clinical situations, and clearly highlight a need for more expansive efforts for fine-tuning of biofluid storage and handling methods, during the important validation of these identified markers that must follow.

### Workshop topic 3. Assays for EV-based markers and rare event analysis

The capacity to identify EV-associated biomarkers in a simple, rapid and cost-effective manner will be a critical step in the development of EV-based biomarkers, and hence the workshop explored some of the potential methodologies to achieve this. Amongst the accepted technologies widely utilised is that of flow cytometry, and Marca Wauben (Utrecht University, Netherlands) presented an overview of the advantages and difficulties of this technology for analysis of EVs.

Critically, flow cytometers were not designed for detection of submicron sized particles, e.g. EVs, and traditional instruments remain limited in their capacity to discriminate EVs from instrument noise. The major issue of multiple small particles providing a single detectable event and how sample dilution can prevent such “swarming” effects, was discussed [27]. Swarm artefacts are also problematic in terms of multi-colour fluorescent labelling and false-positive events. Nevertheless, it is possible to detect rare sub-populations of EVs, 0.01–0.1% of all EVs present, with a well setup instrument and knowing the limitations of the instrument.

To improve the quality of flow cytometric analyses of EVs, an ISEV-ISAC-ISTH EV-Flow cytometry working group is working on consensus of methods, standards and consistent reporting. With respect to reporting, currently most scientific journals do not set criteria regarding flow cytometry experiments, and the working group is preparing a checklist of the most critical details for submission with flow-derived data sets on EVs. Key to the future development of single EV-based flow cytometry would be the involvement of manufacturers in terms of instrumentation design tailored towards EVs. Also, development of calibration beads in the EV size-range and with dim fluorescence as well as reference material with similar physical-chemical properties as EVs are needed.

Joshua Welsh (National Cancer Institute, NIH Bethesda, USA) used flow cytometry of EVs in clinical studies of liver fibrosis, where staining for leukocyte markers on EVs reflected the severity of fibrosis and liver function. Details of why flow cytometer set-up information, gating strategy and the use molecules of equivalent soluble fluorophore (MESF) reference beads and particle scatter modelling were needed for the reporting of EVs in translational studies was explained. The software to convert arbitrary fluorescence units to MESF-units is freely available for FlowJo (http://www.joshuawelsh.co.uk/flowjo-mesf-calculator/), and aids in quantifying aspects such as ligand density on single EVs. Similarly, free software will become available to calculate the EV diameter from light scatter, which should help the community in comparing data between instrument platforms, and help drive improved interoperability, and validation across laboratories.

Carina Levin (Emek MC, Haifa, Israel) discussed the possible utility of EVs as a marker of β-thalassemia major. As well as observing elevations in numbers and size of EVs by nanoparticle tracking analysis, elevated levels of intra-vesicular HSP70 were apparent, which correlated with erythropoiesis and haemolysis parameters.

Benedikt Kirchner (Technical University of Munich, Germany) highlighted that isoforms of miRNA (isomiRs) are rarely examined, yet these may provide useful biomarker utility. Developed tools for isomiR reference file generation and isomiR read count analysis were shown and applied in a study on EVs in clinical depression. Using small RNA sequencing of blood-derived EVs, isomiR analysis increased the robustness of the signal, improving mapping and classification, and 300% more differentially expressed transcripts compared to classical analysis approaches. This analysis can be added to existing datasets/pipelines, and can reveal differences in control, moderate and severely depressed individuals that may not be apparent using traditional tools. These are examples where already-available technology can be adapted for utility in EV measurements and analysis, and reveal biomarker features in different disease settings.

Yoon Kang (Korea Institute of Science and Technology, Seoul, South Korea) used antibody-coated magnetic beads to assess EV-surface proteins, followed by an impedance measurement, configured as a microwell sensor-array [28], to determine ligand binding. This provides a highly sensitive modality for antigen detection, of <1 pg/ml, outperforming ELISA-like platforms. The assay was applied to assess plasma-derived vesicular amyloid β42 in Alzheimer disease, and was capable of identifying late-onset patients compared to controls. This is an example that new EV-detection technology is rapidly evolving, showing good potential enhancement of detection sensitivities with clinical biofluids.

Similarly, George Daaboul (nanoView Diagnostics, Boston, USA, sponsored presentation) discussed affinity immobilisation of EVs on a microarray printed on an engineered chip surface to allow interferometricly enhanced imaging of single vesicles. The instrument requires low input material, 5–100 μL, and provides a label-free size distribution of subpopulations of affinity captured EVs. Although the throughput is currently limited, the platform may present an alternative to flow cytometry for small EVs down to 40 nm. A helpful technology presentation from Clemens Helmbrecht (Particle Metrix GmbH, Meerbusch, Germany; sponsored presentation) described the varied uses of the ZetaView technology in particle sizing, counting and showcased fluorescence and charge-based analyses capabilities of the instrument. In particular, the presentation indicated the awareness of manufacturers of the need for rigour in vesicle analysis, and how factors responsible for variance in the analysis platform can be understood, and minimised.

### Workshop topic 4. Taking EV markers to the clinic

The sample collection processing and assay systems are leading towards real-world utility in clinical settings and there were several successful examples presented pointing to genuine relevance of EVs as clinically useful. A plenary by Jennifer Jones (National Cancer Institute, NIH Bethesda, USA), centred on the ambition to use EVs for personalised medicine, and highlighted that the typical intervals between treatment initiation and measurement of treatment responses span many months, whereas biological effects of treatments are known to occur within days to weeks. Since EVs are released continuously by cells and since those EVs carry biomolecular signatures that reflect the state of the cells that produce them, early post-treatment EV analysis opens a possible window for identification of responses to treatment. Ultimately, it is hoped that early EV analysis may provide a means to determine when treatments are not working and could be adjusted for therapeutic benefit.

Various forms of flow cytometry approaches were presented, including a recent nano-flow (nanoFCM) platform developed by Xiaomei Yan’s laboratory at Xiamen University. This gives superior light scatter-based resolution down into the 40 nm range [29] and single-fluorescent molecule detection and demonstrated identifying EV populations stained for epithelial cell adhesion molecule (EpCAM), prostate-specific membrane antigen (PSMA) and CD147. This next-generation instrument is capable of providing accurate EV concentrations for a specific phenotype, and this quantitation is what clinical laboratories require. Yet this next-generation technology in its current implementation is slow, with sample preparation that is labour intensive and requires research lab- rather than clinical lab-compatible instrumentation to reduce residual fluorophore and other artefacts. Nevertheless, nano-flow sorting offers great potential for detailed sub-population profiling for miRNA content for example, and data of this approach are now emerging from the clinic. Alternative coupling of vesicles to beads, using multiplex bead sets developed by Miltenyi was described, showing improved, multiparametric phenotypic analysis of vesicle subsets than can be achieved with single vesicle cytometric methods. Multiplex analysis also may provide a useful approach to elucidate some of the confounding variables previously discussed around sample collection revealing the loss of certain EV populations dependant on blood tube type, anticoagulant used, etc. There was agreement that issues surrounding collection are highly impactful.

Hidetoshi Tahara (Department of Cellular and Molecular Biology, Hiroshima University, Japan) described the role of miR-22 in regulating cellular senescence, and its application to induce senescence in breast cancer, perturbing growth and metastatic spread. Using a functional high-throughput screening system of senescence-inducible microRNAs, new senescence-associated miRNAs were identified, including one which exhibited high potency in the resolution of tumours in preclinical models. Screening serum or plasma EVs showed some disagreement in the miRNA with more t-RFs (tRNA fragments) in serum, but appeared to be sensitive to detect very small solid cancers (of 5 mm). The study of iso-miRs within such datasets was more informative comparing disease versus controls, than the study of mature miRNAs using an optimised SOP for microRNA analysis from blood. Such analyses were possible with little input material (200 μl), and are therefore likely very compatible with clinical situations.

J. Brian Byrd (University of Michigan, Ann Arbor, USA) stated an unmet need for biomarkers of mineralocortacoid receptor (MR) activation, and questioned whether transcriptional activity (downstream of MR activation) could be detected in urine as a surrogate of MR activity. He described findings from a crossover study of participants with prehypertension who consumed a low-sodium diet and subsequently underwent sodium loading. Potential target transcripts such as those encoding subunits of the amiloride-sensitive epithelial sodium channel and others, were shown to change with sodium loading. In addition, these transcripts correlated directly with serum aldosterone concentrations and inversely with excreted urinary sodium.

Dakota Gustafson (University of Toronto, Canada) discussed relationships between end-stage renal disease, cardiovascular mortality and the current lack of appropriate clinical markers to identify patients at the highest risk of adverse cardiac events. Using high-throughput microfluidic-based qRT PCR on 600 patient plasma-derived EVs he described how miR-125b, miR-23-3p, and miR-124 were identified as possible cardiovascular disease biomarkers and the utility of microfluidics-based EV analysis in the clinic. Additionally emphasised was the clinical overlap between heterogeneous patient groups, in particular those with multiple co-morbidities, and the growing requirement for combinatorial miRNA biomarker panels for robust clinical discrimination.

Andreas Möller (QIMR Berghofer Medical Research Institute, Herston, Australia) described a difficult clinical situation in non-small cell lung cancer (NSCLC), where current pathological testing is insufficient to determine if early-stage patients should undergo therapies in addition to surgery. An assay system was presented, based on the detection of altered protein composition in EVs from transformed NSCLC cells [30]. This might provide a novel biomarker for rational clinical decision-making, stratifying those NSCLC patients most likely to benefit from additional therapies.

Andrew Hoffman (Cummings School of Veterinary Medicine, Tufts University, North Grafton, USA) showcased models from veterinary medicine, highlighting how lessons learned in large mammals (e.g. canine) can equally be applied to patients, particularly as many diseases show striking commonalities with human diseases. Canine mitral valve disease, a model of human mitral valve prolapse and outright prevalent problem in veterinary medicine lacks biomarkers. Studies were presented showing that EV RNA isolation using a commercial isolation kit, exoRNeasy, required 4-fold less plasma volume and shows significantly less variability with low copy number miRNAs compared to ultracentrifugation methods. However, some challenges exist in novel species, where RNAseq data may annotate only well-conserved features. Further, for small animals low volumes of plasma or serum (<0.1 ml) may compel advances in low input isolation, library preparation, sequencing and bioinformatics.

### Workshop take-home messages and future perspectives

The workshop highlighted many exciting areas of EV biomarker research which fully showcase the potential of this field to make a genuine impact on disease identification, predicting disease, tracking responses to therapeutic intervention and personalised medicine. There was a mixture of large replicative studies as well as promising smaller-scale investigations across a variety of disease types.

Among the most prominent issues however was that of the biospecimen, its full provenance, the details of its donor characteristics, environment, collection, storage, transportation and handling prior to arrival at the laboratory for EV analysis. The adage of trash-in equals-trash-out is certainly an issue that should be high on our collective agendas.

Whilst many bio-banking resources have well-established processes for the collection and generation of specimens such as serum/plasma, these protocols have not been developed to consider the preservation of the structural and molecular integrity of EVs and downstream manipulations. It may well be that many of these routinely used by potentially enormous resources, such as the UK Biobank highlighted by Ryan Pink, have a specimen collection that is reasonably compatible with EV studies. If so, this creates many opportunities to define EVs in a normal healthy ageing population as a reference for comparisons with disease subjects. Currently, however, there is a knowledge gap about the variables impacting EVs, and our abilities to perform critical specimen quality assessments prior to embarking on EV investigations.

Collection of blood will introduce peculiar variables compared to urine, cerebrospinal fluid, saliva, milk or other fluids, and as such each type of biofluid brings a new set of unknowns in terms of sample handling. Furthermore, downstream applications will be sensitive to these upstream procedures, such as the use of Polyethylene Glycol precipitants potentially causing issues with mass-spectrometric analyses; this further confounds the development of a single robust sample-handling procedure that suits every need.

How might we identify the parameters that do not have a big effect on EVs and those that indeed do impact EV integrity? It is unlikely for most funding agencies to find a research proposal of this nature sufficiently exciting to warrant investment; yet it is clearly an area which could have enormous future impact. Many studies, in this workshop and published works, have begun to address such questions. It would be useful at this point to collect what is already known to impact features of biofluid EVs (e.g. count, size, integrity, composition and activity), and to identify what consensus exists within our community on such variables, and describe the best practice methods as far as we currently conceive. Value may be gained by scoping other disciplines, such as pharmaceutical liposomes for example and how their presence in biofluids can be maximally preserved. Perhaps, collectively as researchers, we must pay more attention to our laboratory processes, to manage the adherence to SOPs, and to fully document in explicit fashion the details of each step. Usage of EV-TRACK [11] and compliance to the MISEV (minimal requirements for reporting EV-mediated effects) [31] guidelines is encouraged. It is likely that the sharing of quality and well-considered SOPs, even in a background of incomplete knowledge, will immediately aid reproducibility, and allow cross-validation of discoveries to be more successful.

Many delegates agreed that having an ability to physically trace EVs during sample storage/handling procedures would be enormously useful as a tool to hone SOPs. Their nano-scale size makes protocol development difficult because they are so difficult to detect. The idea of a reference EV, EV-like particle or an artificial vesicle-mimic that is fully standardised in terms of numbers/size, etc., perhaps carrying a fluorescent marker was proposed as a means of achieving this kind of quality control. It requires some detailed considerations, however, as the heterogeneity and complexity of EVs will be impossible to fully reflect by an EV reference or artificially prepared EV mimic. Furthermore, we need different types of standards for different applications. Nevertheless, standards including dim-fluorescent beads to aid flow cytometry set up, and facilitate the inter-lab validation of procedures and discoveries will undoubtedly be a forward step in this field. The involvement of companies, with technologies in place to design and certify such standards is likely to accelerate such developments. Similarly, academic researchers and ISEV should collaborate closely with manufacturers, biotechnology companies and regulatory agencies to refine current instruments for EV analysis and to translate EV-based biomarkers into marketable products.

The development of assay systems to measure EVs is an area of rapid growth, both with established platforms like flow cytometry undergoing an evolution towards nano-scale resolution, or other up and coming micro-fluidic/chip array platforms. The factors to consider with all such instrumentation, however, is knowing the mechanism of the platform and its limitations, as without this understanding the data arising may be misinterpreted. Additionally researchers should be aware of input parameters (volumes, buffers and viscosities), limits of detection, linearity, dynamic range, performance time and confounding factors in biofluids that can skew results. Researchers should know the inherent reproducibility and methods of quality control and assurance of the instrument. Validation of rare events could include labelling with different reagents, the use of other complementary assay systems, and to include a spike-in positive control ensuring suitable detection. The presence of nano-aggregates in fluids may provide a signal that is not genuinely related to EVs, and discussions around detergent lysis of EVs may help clarify ambiguities here.

In summary, this ISEV workshop has been a wake-up call for educating EV researchers about the real-world difficulties of biomarker discovery and application. However, we trust sharing information about successes and common confounders in this fashion is a spur for future efforts to overcome, and to bolster the major problem of scientific rigour, reproducibility and transparency that we all face. Funding agencies should also be aware of the enormous socio-economic benefits that EVs research could have for society in general and foster multicentre projects to help to overcome the above mentioned issues. Work groups have been tasked now that will build further on the ISEV position paper on pre-analytical variables [10] with examining in detail what is currently known about pre-analytical variables in relation to blood, urine and other biological fluids. In addition, ISEV will begin the implementation of a standardisation committee in order to fuel accelerated activities, and to progress these aspects of the EV field.

## Workshop Organisers

Aled Clayton (Chair), Cardiff University, UK; David Carter (sponsorship liaison) Oxford Brookes University, UK; Paul Harrison (local host), University of Birmingham, UK; Jason Webber (communications), Cardiff University, UK; Hang Yin, Tsinghua University, China; Charlotte Lawson, RVC, London, UK. ISEV Board members; Chris Gardiner, UCL, UK; Marca Wauben, Utrecht University, NL; Kenneth Witwer, Johns Hopkins, USA; Andrew Hill, La Trobe University, Australia.
